# Evaluation of Restorative Techniques for Vertically Fractured Roots

**DOI:** 10.3390/ma14092099

**Published:** 2021-04-21

**Authors:** Kota Shimizu, Tomonori Satoh, Koichi Shinkai

**Affiliations:** 1 Advanced Operative Dentistry-Endodontics, The Nippon Dental University Graduate School of Life Dentistry at Niigata, Niigata 951-8580, Japan; kota.shimizu@ngt.ndu.ac.jp; 2 Department of Endodontics, The Nippon Dental University School of Life Dentistry at Niigata, Niigata 951-8580, Japan; satu@ngt.ndu.ac.jp; 3 Department of Operative Dentistry, The Nippon Dental University School of Life Dentistry at Niigata, Niigata 951-8580, Japan

**Keywords:** vertical root fracture, bonding durability, microleakage, adhesive restoration

## Abstract

The purpose of this study was to examine the effects of combining specific adhesive materials and various surface treatments on bonding durability and microleakage of vertically fractured roots. Adhesive models were prepared using bovine lower incisors. The experiment included the following five groups: SB-G group (control) (10% citric acid with 3% ferric chloride solution (10-3 solution) + an adhesive resin cement (4-META/MMA-TBB; Super-Bond^®^)), EC group (self-cure bonding agent (UB) + core composite resin (EC)), EC-G group (10-3 solution + UB + EC), EC-P group (40% phosphate solution + UB + EC), and EC-E group (18% ethylenediaminetetraacetic acid (EDTA) solution + UB + EC). After applying a load of 50,000 cycles, microleakage, microtensile bond strength (μTBS), and failure modes were examined. Microleakage of the EC, EC-G, and EC-E groups was significantly lower than that of the EC-P group. The μTBS of the EC-G group was significantly higher than that of the other groups. All EC groups showed that mixed (cohesive and adhesive) and adhesive failures were the most prevalent types of failure modes. The EC-G group showed the highest bonding durability and the lowest microleakage results, which indicates a possible alternative to current adhesive and tooth surface treatments.

## 1. Introduction

Vertical root fractures have become a clinically significant issue in tooth repair. The causes of vertical root fracture include the physical degradation of teeth during cavity preparation and opening of the pulp chamber [1], use of hand files and finger spreaders during endodontic treatments [2,3], and the setting of casting cores after endodontic treatments [4]. In general, a vertical root fracture ultimately results in tooth extraction [5,6].

In recent years, an adhesive restoration method followed by intentional replantation has been reported as a means of repairing teeth with vertically fractured roots. This method consists of tooth extraction with minimal damage to periodontal tissue, removal of the granulated tissue around the root and the root canal filling materials, reconstruction of the fractured root with adhesive material, and intentional replantation [7,8,9]. Some clinical studies reported that ~1 month was required as a temporary splint of a vertically fractured tooth repaired with an adhesive resin [10,11].

In general, an adhesive resin cement based on 4-methacrylicoxyethyltrimerite anhydride/methyl methacrylate-tri-*n*-butylborane (4-META/MMA-TBB, Super-Bond^®^, Sun Medical, Shiga, Japan, SB) is used in the adhesive restoration method [7,8,9,12]. However, disadvantages include poor operability, difficulty in being able to discriminate colors (teeth vs. resin), and long curing times when using SB. Furthermore, it is well-known that cured SB possesses low physical strength and large polymerization shrinkage due to the absence of fillers [13].

Alternatively, the blue composite resin paste for core construction (ESTECORE, Tokuyama Dental, Tokyo, Japan, EC) features facile operability using a hand-type syringe and easy color discrimination. In addition, a chemically cured-type bonding agent (TOKUYAMA UNIVERSAL BOND, Tokuyama Dental, Tokyo, Japan, UB) can easily treat the dentin surface with only blown air when using EC in the adhesive restoration method. Because the combined use of UB and EC appears to be suitable for the adhesive restoration method for reconstruction of vertically fractured roots (short curing time, good handling ability, and easy color discrimination) [14,15], we incorporated this key combination into our experimental protocol.

It has been reported that the type of adhesive material used in the adhesive restoration method affects bonding durability and microleakage [16]; however, the influence of tooth surface treatments such as acid etching and applying self-etching primer has not been evaluated. Therefore, the purpose of this study was to examine the effects of combining specific adhesive materials and surface treatments to evaluate the bonding durability and microleakage of vertically fractured roots in the adhesive model. The null hypothesis was that the adhesive materials and the surface treatments would not affect the microtensile bond strength (μTBS) or the microleakage of repaired vertically fractured roots.

## 2. Materials and Methods

**Figure 1:**
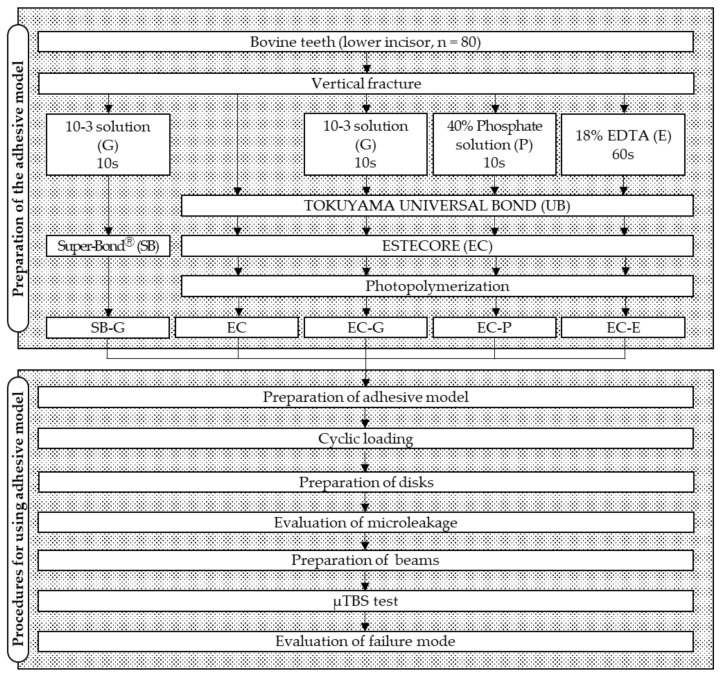
Flowchart of the experimental procedure. 10-3 solution, 10% citric acid with 3% ferric chloride solution; EDTA, ethylenediaminetetraacetic acid; µTBS, microtensile bond strength.

The flowchart of the experimental design is shown in Figure 1.

### 2.1. Experimental Groups

We used power analysis software (G*Power 3.1.9.7) to determine the appropriate number of specimens for statistical analysis of the five groups. A priori power analysis showed that 16 specimens would be required in each group to achieve a 0.8 significance power, an alpha error probability of 0.05, an effect size of 0.4, and five groups for one-way ANOVAs. A total of 80 lower incisor bovine teeth were used for this study.

Five experimental groups were set up with combinations of adhesive and tooth surface treatments, as shown in Table 1. The experimental groups were divided into two groups: one used conventional SB and the other used EC. The EC groups were further divided into four groups: one used only UB, whereas the others used tooth surface treatment materials commonly used in clinical practice.

### 2.2. Preparation of the Adhesive Model of Vertically Fractured Roots

The adhesive models of vertically fractured roots were prepared with reference to a previous study [16]. Using a low-speed diamond saw with a 300 µm blade thickness (IsoMet^TM^, Buehler, Lake Bluff, IL, USA), the bovine teeth were horizontally cut at the cementoenamel junction to remove the crown. The dentin around the root canal was removed using a diamond drill (DHP 2021 #120, Argo file, Tokyo, Japan) until the thickness of the root canal wall was 1.5 mm. After the initial preparation, the roots were cut longitudinally and mesio-distally using the IsoMet^TM^ diamond saw to a depth of 17 mm from the coronal. This created a 300 μm gap available for incorporation of the adhesive materials. Then, the remaining apical parts were divided longitudinally.

**Figure 2:**
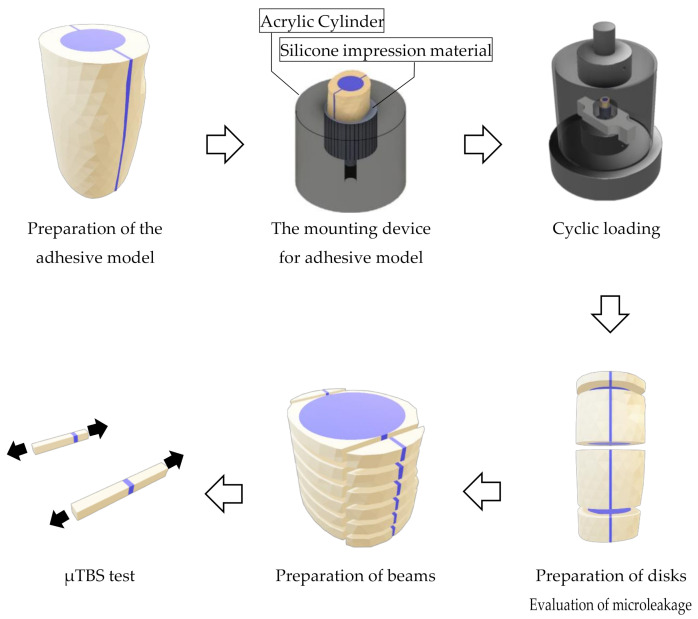
Procedures for using the adhesive model.

Each cut fragment surface was treated with solutions according to the conditions of each experimental group as shown in Figure 1. The 10-3 solution and phosphate solutions were applied for 10 s, and EDTA was applied for 60 s followed by rinsing and drying. After these teeth surface treatments, UB was applied to the specimens in the EC groups, and the fragments were restored. In the EC groups, the fragments applied with EC were repositioned, and then EC was photopolymerized using a light curing unit (PenCure 2000, J. Morita, Kyoto, Japan). In the SB-G group, SB was applied to the fragments, and then they were repositioned. After repositioning, the excess adhesive material was removed. When repositioning the fragments, the divided apical parts were used as a guide. After storing the restored fragments in 37 °C distilled water for 24 h, the apical parts were removed to create the 17 mm adhesive models (Figure 2).

### 2.3. Cyclic Loading for the Specimens

A mounting device for the specimens, which has a socket (10 mm in diameter) and a spillway (2.7 mm in diameter) at the bottom, was fabricated using an acrylic cylinder (20 mm in height, 25 mm in diameter). When mounting the specimen, the socket was filled using a mixed silicone impression material (EXAFINE Putty Type, GC, Tokyo, Japan), and then the specimen was inserted into the center of the socket until the silicone impression material hardened. The acrylic sockets and the hardened silicone impression materials simulated alveolar bone and periodontal membranes, respectively (Figure 2).

The specimens mounted in the device were repeatedly challenged with a cyclic load (70 N, 2 Hz) in 37 °C water using a dynamic fatigue tester (ElectroPuls^®^ E3000NL, Instron^®^, Norwood, MA, USA) for 50,000 cycles, which simulated a 1 month mastication time period. The dynamic loads were directed onto the flat surface of the specimens using the stainless cylinder (50 mm in diameter) (Figure 2). After the cyclic loading tests, the specimens were stored in 37 °C water for 24 h.

### 2.4. Evaluation of Microleakage

To evaluate microleakage, the specimens were immersed in a 50% silver nitrate solution (0.02 mol/L-Silver Nitrate Solution, Nacalai Tesque, Kyoto, Japan) in a darkroom for 24 h. After washing the specimens under running water, they were immersed in X-ray developer (GBX developer, Care Stream Dental, Atlanta, GA, USA) and exposed to fluorescent light for 8 h. After washing and drying the specimens, they were horizontally cut at 1, 6.5, and 13 mm from the coronal side to obtain three disks per specimen (Figure 2). Both sides of each disk were observed using a stereomicroscope (EZ40D, Leica Microsystems, Wetzler, Germany) at 12.5× magnification. We used 16 bovine teeth to prepare the specimens for each group. For the microleakage test, three disks were prepared per specimen and both sides of disk were observed, resulting in 32 data points for each group at each cut position (1, 6.5, and 13 mm from the coronal side).

Disk microleakage was evaluated on the basis of four classifications [17]:

Class 1: No staining,
Class 2: Staining of <50% of the tooth thickness along the fractured line,
Class 3: Staining of >50% of the tooth thickness along the fractured line,
Class 4: Staining reached the root canal wall.

### 2.5. Microtensile Bond Strength (μTBS) Tests

Specimens were cut horizontally to obtain ten 1 mm thick disks using the IsoMet^TM^ saw. Bonded areas positioned at the mesial and distal parts of each disk were cut vertically to prepare 20 beams with an adhesive area of 1 mm^2^ (Figure 2). The mean value of the μTBS data obtained from the 20 beams represented the μTBS value for a specimen. Using a model repair agent (Model Repair 2, Dentsply Sirona, Tokyo, Japan), these beams were attached to a testing device (BENCOR Multi-T, Danville Engineering, San Ramon, CA, USA). This device was placed on a compact table-top material tester (EZ-Test, Shimadzu Corporation, Kyoto, Japan) and was subjected to the μTBS test at a crosshead speed of 1 mm/min. For the microtensile bond strength test, 20 beams were prepared per specimen and were used to obtain the mean bond strengths value of each specimen, resulting in 16 data points obtained for each group.

### 2.6. Evaluation of Failure Mode

After the µTBS test, the fractured surfaces of the beams were observed using a stereomicroscope (Leica EZ40D) (35×). Failure modes were classified into four types [18]:

Type 1: Adhesive failure (>80% of failures occurred at the interface between the adhesive material and the dentin),
Type 2: Cohesive failure in adhesive material (>80% of failures occurred within the adhesive material),
Type 3: Cohesive failure in dentin (>80% of failures occurred within the dentin),
Type 4: Mixed failure (adhesive and cohesive mixed failures).

In addition, the fractured surfaces of the representative specimens with typical failure modes in each group were observed with a scanning electron microscope (SEM) (Miniscope^®^ TM4000Plus, Hitachi High-Tech Fielding, Tokyo, Japan).

### 2.7. Statistical Analysis

The Bell Curve for Excel version 3.20 (Social Survey Research Information, Tokyo, Japan) was used for statistical analyses. Microleakage data for each group were statistically analyzed using the Kruskal–Wallis test followed by the Steel–Dwass post hoc test. The μTBS data for each group were statistically analyzed using one-way analysis of variance (ANOVA), followed by the Tukey post hoc test. The confidence level was 95% (α = 0.05).

## 3. Results

The results of the Kruskal–Wallis test for the microleakage test showed no significant differences among the cut positions of the specimens in each group (*p* > 0.05), whereas significant differences were detected among the groups for each cut position of the specimens (*p* < 0.001). The Steel–Dwass post hoc test revealed that the distribution of the microleakage score for the EC-P group was significantly different from those for the other EC groups at 6.5 mm from the coronal cutting surface (*p* = 0.0227) (Table 2). There was no significant difference in the distribution of microleakage scores at 1 mm and 13 mm from the coronal cutting surface.

**Figure 3:**
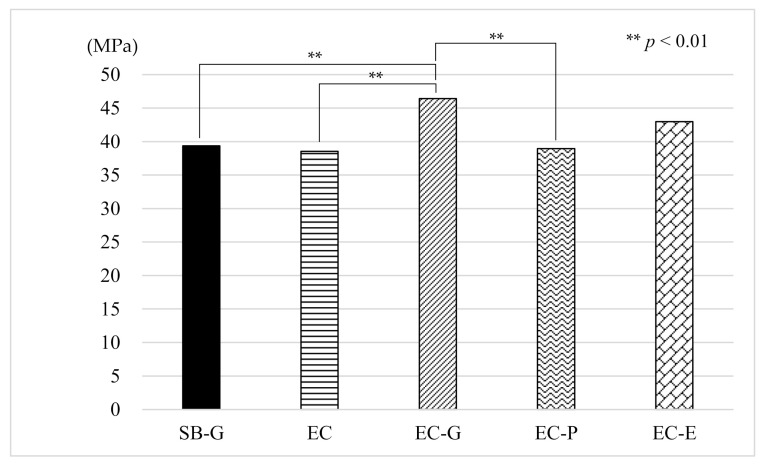
Results of the µTBS measurements. µTBS: microtensile bond strength, SB: Super-Bond^®^, EC: ESTECORE, G: 10-3 solution, P: phosphate solution, E: ethylenediaminetetraacetic acid, MPa: megapascal.

The mean μTBS of the EC-G group was the highest (46.43 MPa) among all the EC groups with the SB-G group (control) being 39.36 MPa. The results of the one-way ANOVA for the μTBS tests showed significant differences among the groups (*p* < 0.001). The Tukey post hoc test revealed that the μTBS from the EC-G group was significantly higher than that of the SB-G group (*p* = 0.0092), EC group (*p* = 0.0027), and EC-P group (*p* = 0.0051) (Figure 3).

**Figure 4:**
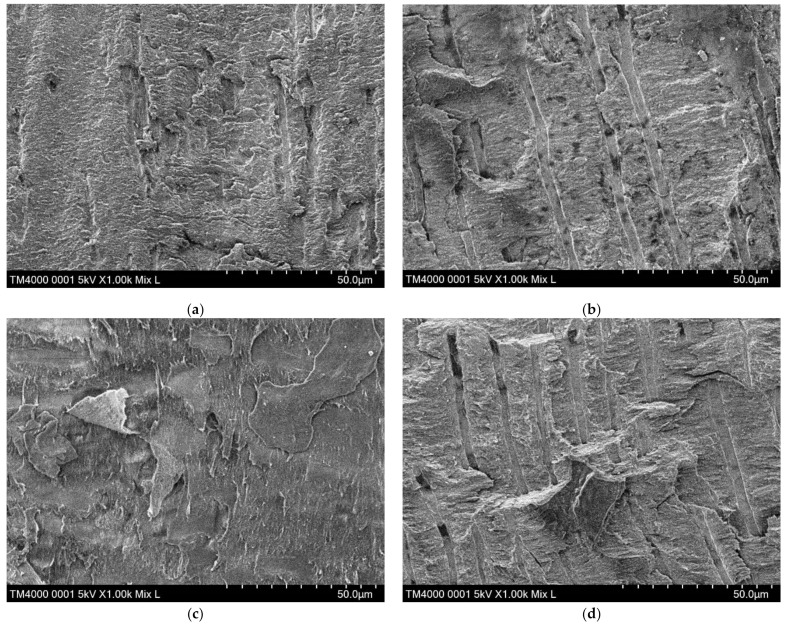
Representative SEMs of typical failure modes in each of the EC groups. (**a**) EC group: the dentin surface is covered with a smear layer and a few unclear dentinal tubules; (**b**) EC-G group: the dentinal tubules are clearly observed; (**c**) EC-P group: the structure of the dentin surface is amorphous; (**d**) EC-E group: the dentinal tubules are clearly observed. SEM, scanning electron microscope; EC, ESTECORE.

In the SB-G control group, the prevalence of the specimens showing type 1 adhesive failures and type 2 cohesive failures dominated (57.14%, and 34.07%, respectively). Among the EC groups, the distribution of failure modes was characteristic depending on tooth surface treatment materials. The EC group showed the highest prevalence of the type 4 mixed failure, whereas groups SB-G, EC-G, EC-P, and EC-E exhibited those of the type 1 adhesive failure. The EC-G group revealed the highest prevalence of the type 3 cohesive failure (Table 3). Representative SEM micrographs of typical failure modes in each of the EC groups are shown in Figure 4.

## 4. Discussion

The results of this study showed that the bonding durability and microleakage of the adhesive models of vertically fractured roots were affected by the types of material and tooth surface treatments; hence, the null hypothesis was rejected. Recently, it has been difficult to obtain large numbers of extracted human teeth due to the decrease in tooth extraction procedures and due to ethical issues. Therefore, in this study, bovine teeth were used instead of human teeth, which increased the importance of establishing the similarity between human and bovine teeth. Several studies reported the comparison of human and bovine teeth from different points of view. One study, which evaluated the structural and morphological differences between human and bovine root canals, reported that the tubule densities of radicular bovine teeth were significantly higher than that of human teeth, whereas significant differences in the diameters of the tubules were not detected [19]. Another study, which examined the number and diameter of dentin tubules in root canals of human and bovine teeth, reported similar results [20]. A review article that examined whether bovine teeth could be substituted for human teeth in dental research concluded that bond strength test results were inconclusive when using bovine teeth as an alternative to human teeth [21]. However, it is important to note that, other than bovine teeth, no alternative substitutes for human teeth exist.

In this study, the EC-G and EC-E groups showed high mean µTBS values. This may be because of effective and sufficient resin-impregnated layer formations by UB applications on bonding durability and stability [22,23]. Furthermore, the tooth surface treatments using 10-3 solutions and EDTA were more effective compared with phosphate solution in removing contaminants and thin layers of cutting debris (smear layers) without excess dentin decalcification [24,25,26], which may have improved the bond strength. When performing adhesive restoration methods for vertically fractured roots followed by intentional replantation in a clinical setting, blood contamination for the fractured root surface is assumed. However, a previous study reported that the µTBS of the contaminated dentin after treatment with 10-3 solution was equivalent to that of noncontaminated dentin [24]. Therefore, using 10-3 solution may be beneficial in adhesive restoration methods.

The results of the microleakage statistical analyses in this study showed no effect due to the adhesive material; however, rather significant effects due to tooth surface treatments were observed. The lack of significant difference in microleakages between the materials may be because polymerized chemically cured-type resins, such as SB, show low contraction stress despite large polymerization shrinkage [27]. The results of this study showed that pretreatment with the 10-3 solution or EDTA was more effective in suppressing microleakage compared with a phosphate solution, which is consistent with previous reports [28]. Microleakage tests can identify the existence of contraction gaps between teeth matrices and restorative materials [29]. The formation of gaps is created by low bond strength and large polymerization shrinkage of the restorative material [30]. Hence, the excellent marginal sealing shown in EC groups with the exception of the EC-P group might be due to the high bond strength and small polymerization shrinkage of the adhesive system or material applied. Because bacteria living in these gaps may affect periodontal tissue [12], the pretreatment using 10-3 solution or EDTA may be advantageous in this adhesive restoration method.

In previous reports concerning adhesions on the dentin surface [31,32], removal of the smear layer and the formation of resin tags were evaluated. In this study, many longitudinally cut dentinal tubules were observed on the SEM images of the dentin surface in the EC-G and EC-E groups. Because these tubules seem to be useful for increasing adhesion areas, the removal of the smear layer covering on the cut dentin surface may become more important for adhesion in vertically fractured roots. The failure modes observed in the EC-G group were unique in the five experimental groups because there were fewer adhesive failures and more cohesive failures in dentin compared with other experimental groups. The failure mode characteristics shown in the EC-G group tend to occur in cases of high bond strength between dentin and adhesion materials [33].

The adhesive materials used in this study (ESTECORE) contain several resin monomers such as Bis-GMA, Bis- MPEPP, and TEGDMA. Previous studies reported that unpolymerized resin monomers exhibited fibroblast cytotoxicity [34,35,36]. However, the cytotoxicity of the resin monomers was dependent on the degree of conversion of polymerized resin composites. The polymerized resin composite removed the oxygen-inhibition layer and showed significantly lower cytotoxicity than the oxygen-inhibition layer [36]. Therefore, we considered that the polymerized adhesive materials could be used safely for periodontal tissues during the reconstruction of fractured roots, although it will be necessary to prove the biocompatibility of these adhesive materials to periodontal tissue cells in the future.

From the results of this study, it was concluded that EC is a suitable adhesive system as an adhesive restoration method of vertically fractured roots. The EC-G group, wherein the vertically fractured root was repaired with UB and EC after applying the 10-3 solution, showed the highest bonding durability and the lowest microleakage in the cyclic loading test replicating 1 month of mastication during placement in the temporary splint. Therefore, the adhesive restoration method for vertically fractured roots used in this study demonstrated bonding durability sufficient for 1 month of mastication.

The results of this bonding durability study of reconstructed fractured roots under cyclic loading tests simulated only short-term temporary splints. Hence, the potential of this bonding durability reconstruction method for fractured roots over the long term is still unclear. Moreover, while simulating clinical conditions, it would be necessary to examine the efficacy of this adhesive method for the reconstruction of fractured roots with cores and crowns.

## 5. Conclusions

The EC-G group, wherein the vertically fractured roots were repaired with UB and EC after applying 10-3 solution, showed the highest bonding durability and the lowest microleakage following a cyclic loading test.

## Figures and Tables

**Table 1:** Experimental groups in this study.

Group	Tooth Surface Treatment	Adhesive Material	Lot; Composition
SB-G (control, *n* = 16)	10-3 solution	Super-Bond^®^ Powder liquid catalyst	TG2; PMMA SV2; MMA, 4-META SS41; TBB
EC (*n* = 16)	UB	ESTECORE	HB301B1; Bis-GMA, TEGDMA, Bis-MPEPP, silica zirconia filler, camphor quinone, radical amplifiers, peroxide, others
EC-G (*n* = 16)	10-3 solution + UB	ESTECORE	
EC-P (*n* = 16)	40% phosphate solution + UB	ESTECORE	
EC-E (*n* = 16)	18% EDTA + UB	ESTECORE	

10-3 solution, 10% citric acid with 3% ferric chloride solution (Super-Bond^®^ Green Activator, Sun Medical, Shiga, Japan); UB, Chemically cured-type bonding agent (TOKUYAMA UNIVERSAL BOND, Tokuyama Dental, Tokyo, Japan); 40% phosphate solution, K-etchant GEL, Kuraray Noritake Dental, Tokyo, Japan; EDTA, ULTRADENT EDTA 18%, Ultradent, South Jordan, UT, USA; 4-META/MMA-TBB, 4-methacrylicoxyethyltrimerite anhydride/methyl methacrylate-tri-*n*-butylborane.

**Table 2:** Microleakage results at each cut position in the adhesive model.

Cut Position	Group	Class 1	Class 2	Class 3	Class 4
1 mm	SB-G	30	2	0	0
	EC	32	0	0	0
	EC-G	32	0	0	0
	EC-P	28	4	0	0
	EC-E	32	0	0	0
6.5 mm	SB-G	29	3	0	0
	EC	32	0	0	0
	EC-G	32	0	0	0
	EC-P *	24	8	0	0
	EC-E	32	0	0	0
13 mm	SB-G	26	6	0	0
	EC	32	0	0	0
	EC-G	31	1	0	0
	EC-P	28	4	0	0
	EC-E	32	0	0	0

SB, Super-Bond^®^; EC, ESTECORE; G, 10-3 solution; P, phosphate solution; E, ethylenediaminetetraacetic acid; * The microleakage score of EC-P group was significantly higher than those of the other groups at the cut position of 6.5 mm.

**Table 3:** Results of the failure mode analyses.

Group	Failure Mode (%)			
	Type 1	Type 2	Type 3	Type 4
SB-G	57.14	34.07	0.73	8.06
EC	31.71	24.39	1.39	42.51
EC-G	42.00	19.00	9.67	29.33
EC-P	88.77	3.51	0.00	7.72
EC-E	54.63	9.58	1.28	34.50

SB, Super-Bond^®^; EC, ESTECORE; G, 10-3 solution; P, phosphate solution; E, ethylenediaminetetraacetic acid; Type 1, adhesive failure; Type 2, cohesive failure in adhesive material; Type 3, cohesive failure in dentin; Type 4, mixed failure.

## Data Availability

The data presented in this study are available on request from the corresponding author.
